# The Integration of Social Science for Community Engagement in the Humanitarian Fields of Conflicts and Disasters: A Scoping Review

**DOI:** 10.3390/ijerph20196856

**Published:** 2023-09-28

**Authors:** Luisa Toro-Alzate, Paola Maffi, Anu Puri, Rania Elessawi, Maria Falero Cusano, Jozefien Groenendijk, Daniel H. de Vries

**Affiliations:** 1 Amsterdam Institute for Global Health and Development (AIGHD), 1105 BP Amsterdam, The Netherlands; l.toro-alzate@aighd.org; 2 Athena Institute, VU University, 1081 HV Amsterdam, The Netherlands; p.maffi@vu.nl (P.M.);; 3 UNICEF Europe and Central Asian, 1211 Geneva, Switzerland; apuri@unicef.org; 4 UNICEF NY Headquarters, Social Behavior Change, New York, NY 10017, USA; relessawi@unicef.org (R.E.);; 5 Department of Anthropology, University of Amsterdam, 1018 WV Amsterdam, The Netherlands

**Keywords:** community engagement, social sciences, humanitarian action, conflict, hazards, disasters

## Abstract

Community engagement (CE) is essential to humanitarian assistance, and the social sciences have been credited in recent epidemics and disease outbreaks as having played a crucial, supportive role. Broadening this attention to other humanitarian fields, this scoping review asks what lessons learned can be found in grey and peer-reviewed literature on the integration of the social sciences in CE for conflicts and disasters. Using an analytical framework developed through a UNICEF-led project called Social Science for Community Engagement (SS4CE) in Humanitarian Action, we identified 1093 peer reviewed publications and 315 grey literature reports of possible relevance. The results show that only a small minority—18 publications and 4 reports—tangibly comment on the relevance of social sciences, mostly only in passing and implicitly. While social science techniques are used and the importance of understanding a community’s cultural, linguistic, and religious context is emphasized, further discussion on the integration of transdisciplinary and multidisciplinary social sciences is absent. Furthermore, CE is mostly seen as an instrumental (‘means to an end’) involvement, for example to collect data in emergency situations and receive feedback on interventions, but not as a critical and transformative intervention. We conclude that unlike the attention given to social sciences in disease outbreaks, there is a knowledge gap and an accordingly proper planning and implementation gap regarding the potentiality of social science to improve CE across all humanitarian contexts of disasters and conflicts.

## 1. Introduction

Humanitarian crises, including disease outbreaks, conflicts, or disasters, have been continuously increasing in the last years. More than 1 percent of the world’s population is now displaced, of which 42 percent are children [1]. Political conflicts continue compromising civilians, especially in LMIC [1,2]. Community engagement, the approach to support communities in taking their own action to address their own pressing needs, is essential in humanitarian assistance [3]. While having played an important historical role in advocating for community rights during the HIV/AIDS crisis, in recent decades, the social sciences—in particular medical anthropology, sociology, and health psychology—have increasingly been highlighted as important to the management of disease outbreaks and epidemics for their assistance in community engagement, since the 2014–2015 west African Ebola outbreak. Social scientists have provided crucial support in humanitarian program design, interpretation, investigation and response, event analysis, and post hoc assessments [4,5]. The use of social sciences in the engagement of local communities and affected populations in the humanitarian response is said to enable community trust and helps to address the highest community priorities sensitively [2]. However, while there is increasing evidence of the value of social science in community engagement during public health emergencies, in the other humanitarian areas of conflicts and disasters, this attention has been much less pronounced [3,5,6]. What lessons learned and best practices have been identified in these particular humanitarian contexts?

This scoping review aims to identify the lessons learned and best practices on the relevance and integration of the social sciences for community engagement in humanitarian action (preparedness, response, and recovery) of conflicts and disasters described in the grey and peer-reviewed literature. Social sciences are defined as generally providing an understanding of structures power, processes, beliefs, norms, attitudes, and behaviors in different contexts. In this review, social sciences are defined as any branch of academic study or science that deals with human behavior in its social and cultural aspects. We mostly looked at anthropology, sociology, health psychology, political sciences, and post-disaster social entrepreneurship. The review was foundational to delivering a core pillar of for the Social Sciences for Community Engagement in Humanitarian Action (SS4CE in HA) project led by UNICEF, with funding from the U.S. Bureau of Humanitarian Assistance, during 2021–2022. The scoping review further informed the identification of capacity needs and gaps, the development of a database, and the analysis of existing SS4CE trainings as well as a competency framework on integrating social science approaches for CE, primarily in conflict and disaster settings. This work was conducted by Sonar-Global’s partner, the Amsterdam Institute for Global Health and Development (AIGHD) with support from Institut Pasteur. AIGHD and UNICEF co-chaired a technical working group (TWG2) throughout the implementation phase of this project and facilitated a co-constructive dialogue and consultation process through meetings and interviews. The deliverables of this project are aimed at humanitarian practitioners and programmers at all levels (e.g., field, senior, HQ), donors, and social scientists working in applied humanitarian as well as academic settings. It intends to further inform the process of integrating and mainstreaming the social sciences in CE in HA.

## 2. Materials and Methods

This scoping review was conducted on both the published and grey literature (reports, papers, and other unpublished documents) generated by practitioners in the humanitarian field. The methodology was developed by a research team consisting of social scientists from the Amsterdam Institute for Global Health and Development (AIGHD) and shared and approved by the Capacity Development Technical Working Group-2 (TWG2) on capacity development as part of a broader UNICEF-led SS4CE in HA project. The methodology was based on a framework proposed by Arksey et al. [7] and guided by the Joanna Briggs Institute (JBI) manual for scoping reviews [8] and the preferred reporting items for systematic reviews and meta-analysis (PRISMA) checklist for scoping reviews [9]. A review protocol was developed to reduce bias and ensure the quality of the review, but this protocol was not registered. 

Key elements from the research question were structured using the population, concept, and context (PCC) design to guide the search query formulation. The PCC framework is recommended by the JBI manual and was helpful in clearly illustrating the focus of this scoping review [8]. Table 1 shows the PCC framework used in this scoping review.

The key concepts established in the PCC framework were converted into keywords to guide the search strategy. The keywords included social sciences, humanitarian response, hazard/disaster, conflict, and community engagement. To expand the scope of the search, and due to its relevance in the humanitarian field, specific terms involving capacity development in the humanitarian field were added, such as skills/competencies for social sciences, capacity needs assessments, and humanitarian practitioners/organizations. 

A systematic search for published literature articles was performed in the electronic databases Web of Science, Scopus, CINAHL, and APA PsycInfo. These databases were chosen for their broad scope and international validity. The search string used to retrieve articles included different types of social sciences; the keywords used to include these were: “Social science*” OR “Social Scientist*” OR “anthropolog*” OR “sociolog*” OR “psycholog*” OR “global health” OR “international public health” OR “global health research*” OR “social research*” OR “health research*” OR “social researcher*” OR “political science*” OR “politics”. In a second complementary round, an additional search on post-disaster social entrepreneurship was conducted and the findings added. The search was limited to a year span of 23 years, from 2000 to 2023, to ensure the retrieval of relevant and updated evidence. The final search was performed on the 30 June 2023. All articles retrieved were then uploaded into the electronic citation manager Rayyan. This database allowed for the screening of eligible articles and the removal of duplicates. 

To complement the search for published literature articles, a systematic search in grey literature databases was conducted with a similar year span of 23 years (2000–2023) to ensure up-to-date evidence based on humanitarian practice. In addition, direct contact with stakeholders working in the field was made to retrieve any grey literature articles they could provide (reports, evaluations, and/or capacity assessments), as not all of the grey literature is published online.

Two team members screened peer-reviewed, published articles independently for eligibility, initially based on titles and abstracts. The screening procedure was piloted collaboratively on a sample of ten articles to ensure the integrity of the screening process. Furthermore, cross-checking was conducted to solve uncertainties and discrepancies. Secondly, articles were selected based on a full-content assessment. The selection of primary and secondary articles was conducted based on pre-established criteria. Articles were considered eligible if they concerned the social sciences in the humanitarian context of response, risk mitigation, and preparedness to natural disasters or conflicts. Only peer-reviewed articles and primary and secondary articles were included to ensure the evidence’s quality. Finally, only studies that were published in English and from the last 23 years were included to ensure that recent and up-to-date evidence on humanitarian practices was included. Studies explicitly focusing on the involvement of social scientists in the COVID-19 pandemic or other pandemics (i.e., Ebola) were excluded from the search. Studies addressing different problems (apart from disaster and conflict management), just community management, or background articles were also excluded.

After removing duplicates, the total number of peer-reviewed articles retrieved from the databases was eight hundred sixty-one. After the review of the titles and abstracts, eighty peer-reviewed published articles were selected for full-text screening, and twenty-one of them met our selection criteria. Of these, ten articles were retrieved through snowballing of the reference lists. All the studies that did not meet the criteria of social science research, community engagement, or humanitarian crises (limited to natural disasters or conflict) were excluded. Studies that referred to epidemic preparedness, for example, epidemic response to Ebola, health crises related to specific diseases (i.e., HIV), studies that did not mention (social science) research, or applied social science research, were excluded.

**Figure 1:**
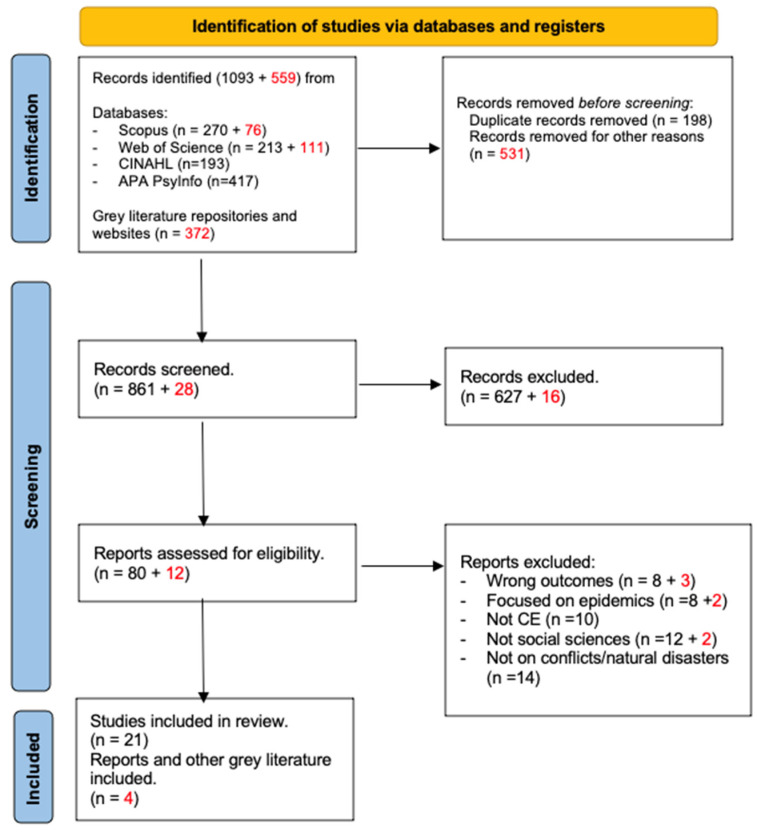
PRISMA flowchart of published and grey literature (numbers in red belong to grey literature) PR: peer-review literature, GL: grey literature.

The initial preliminary findings were presented and discussed with the Capacity Development TWG2, and feedback was received on the process and content. One key recommendation from that consultation was to include a grey literature review. In the next phase, in addition to the published literature, the grey literature was screened based initially on the title and abstract and then in a full-content assessment based on the pre-established criteria. The total number of documents retrieved from the databases was five hundred fifty-nine. From this initial retrieval, the team excluded documents unrelated to conflict and natural disasters or related to epidemic preparedness and response. Also, the team excluded trainings and training guidelines. As a result of this, twelve reports were selected based on their title and abstract, but only four met the selection criteria. The articles selection is graphically displayed with the PRISMA flowchart in Figure 1. A full overview of the selected studies, peer-reviewed, and grey literature can be found in Table S1 (Supplementary Materials)

Relevant data were extracted from the selected documents using a data extraction sheet in Excel. The initial extraction categories were based on the results from the initial phases of the UNICEF SS4CE in HA project. Relevant information was extracted from the findings with an iterative extraction process. One team member extracted data from the articles, and another reviewed the data and evaluated their relevance. After that, the whole team resolved discrepancies and doubts through discussion. No additional extraction categories were added after the review of the manuscripts, as no new themes emerged.

After all the data were input into the data extraction sheet, the team integrated the findings from the peer-reviewed and grey literature. The analysis was conducted by two team members with consultation and support from the third person. Quality checks were performed every week. 

## 3. Results

### 3.1. Widespread (But Implicit) Use of Social Science Methodologies for CE in Humanitarian Contexts

The results indicate that social science approaches can ensure the participation of communities, thereby improving the sustainability of the interventions. The methods used in the social sciences were shown to increase community resilience through more inclusive consultation procedures that acknowledge the experience of participants and give everyone a chance to voice their opinion [10]. In addition, CE was portrayed as effective in delivering interventions that are truly culturally acceptable [11]. However, most of the articles and reports selected for review did not explicitly mention that the used approaches were part of the social science sphere, even though many were easily traceable back to social science roots. For instance, social science research methods are regularly used to develop and evaluate community engagement practices in studied populations (e.g., focus group discussions, key informant interviews, and perception surveys). The use of social science approaches and qualitative research was recognized as a tool to understand the patterns seen in the quantitative data findings [12]. Qualitative research methods also enabled participants to share their experiences, concerns, and queries, providing an additional layer of understanding, which can be used for designing tailored community engagement strategies and interventions [12,13]. Box 1 shows examples from the literature.

Box 1 **Examples of social-science-informed approaches supporting CE.**

**Cultural and needs assessments:** Social science techniques such as cultural and needs assessments were presented as essential for community engagement in disaster settings. Song et al. (2013) emphasized the need of conducting a cultural assessment to build a knowledge base about the country and find local partners that can help researchers learn about local communities and culture, even before executing a need assessment [14]. The latter is often utilized to map the available community resources as well as the mental health and psychosocial needs of the community [13,14].

**Participatory community approaches:** Several participatory community approaches were highlighted in the reviewed literature. For example, Lee (2008) used a rapid participatory approach to gather beneficiary feedback and inform program planning [15]. O’Sullivan et al. (2014) used a community-based participatory research design, which focused on partnerships, engagement, co-learning, and building on existing assets within a community [12]. Specifically, they used the structured interview matrix (SIM) as a facilitation method to engage organizations and citizens in a mapping exercise to identify resources and functional needs [12]. Karadag et al. (2021) conducted research using a participatory community approach, highlighting that the quality of research involving migrants and refugees is improved by using social-science-derived methods that involve collaboration with community leaders, cultural mediators, and civil society organizations as well as peer-to-peer methodologies for data collection, analysis, and reporting [16]. Time and effort dedicated to respect locals’ norms and leadership ensure increased participation and trust [11]. In addition, Durrance-Bagale et al. (2022) emphasized that social science methods can ensure that CE approaches empower communities rather than merely being a consultative process. Therefore, adopting a relational approach instead of a transactional one. They can also move from a transactional to a relational approach [11].

**Collective approach:** Although social science approaches were not directly mentioned, Barbelet (2020) highlighted how focus group discussions provide a “collective approach” to community engagement in conflict areas, which is valuable and complementary to other individual approaches [17] and an effective way to obtain feedback from the population and to feed the existing coordination and decision-making mechanisms [16,17]. In this approach, communication channels, either “people-oriented” or through communication technologies, are emphasized [17]. Community feedback is highlighted as a way to strengthen the engagement of the community in the humanitarian context and allow for more analysis of significant trends in the community [16,18]. In the central Sulawesi response, for example, data collected through such feedback led to a leaflet named “Suara Kumonitas”, which gave voice to the community in high-level decision-making processes [18].

**Local capabilities approach:** The capacity-building model employed by Rivera-Holguín et al. (2016) is another methodology relevant to the process of social reconstruction, which has close links to social science approaches [19]. This model is based on the identification of local capabilities, the development of a shared language, and the implementation of a group process, using workshops and art to strengthen members’ cultures and identities [20]. The end goal is a systematic rise in community leadership and a gradual withdrawal of outside experts [20]. Cueto et al. (2015) also used a methodology frequently utilized in social science called participatory action investigation (PAI) [21]. PAI views community members as social actors with the capacity to make decisions, reflect, and actively engage in the analysis of issues that directly impact them and in the alteration of realities that call for transformation [22]. In an example reported by Durrance-Bagale et al. (2022), community-based health programs in South Sudan and Haiti engaged service-users in decision-making, and therefore community leaders and members were reassured of mental health program benefits, and traditional healers were not threatened as all were involved in the goal setting [11]. Lastly, Panter-Brick (2022) worked on de-constructing the institutional culture that viewed the host and refugee populations, in conflict-affected areas, as research implementers rather than co-creators of evidence aiming to achieve empowerment and social and structural change [22].

**Direct delivery of intervention by community members:** Finally, the role of social science techniques in the direct delivery of interventions by community members is illustrated in the study of Padmavati et al. (2020), where local community workers (CLWs) were trained to expand rehabilitation possibilities and ensure long-term disaster management when incoming aid inevitably leaves [23]. Also, in the study of Tuerk et al. (2013), the intervention included the incorporation of members of the cultural group being trained as actual trainers and planning partners, which has been hypothesized to be an important component in post-disaster mental health group interventions [24]. All throughout these processes, the literature emphasizes the importance of seeing the community as true partners in collectively documenting and voicing their experiences, which fosters a culture of a mutually supportive research environment [24].

Overall, the reviewed literature emphasizes the importance of understanding the community’s cultural, linguistic, and religious context in humanitarian emergencies of conflict and disasters and, as shown, we saw several social science techniques being used [13]. However, despite this widespread use, this same literature is rather silent on the broader relevance of social science knowledge and its potential for broader, more widespread critiques on societal structures and systems that come from anthropological or sociological insights into culture or political insights into power dynamics. This broad literature search did not find any clear distinction between different social science disciplines and their use for community engagement. As such, a more detailed analysis of the contribution of each discipline and the various ramifications of these did not seem needed. Moreover, just one of the documents reported the use of social sciences professionals or trained experts to tailor the community engagement process to the local context. In this respect, Panter-Brick (2022) mentioned the use of biocultural research; more specifically, part of this focuses on “vertical slice ethnography”, which consists of generating relevant data to knit together communities, science, and practice (and policy) [22]. But in most of the articles, for example, in the central Sulawesi response, most members of the working groups from the humanitarian organization were not involved nor trained in social science approaches, and there were only a few people with dedicated roles in communication, community engagement, and accountability [19].

### 3.2. Complexities around the Integration of Social Sciences in the Humanitarian System

The reviewed literature highlighted the importance of effective collaboration between different actors, both outsider groups such as international organizations and insider groups such as local communities [25,26]. Improved collaboration between individuals in the field and those in the academic environment was noted to promote an appropriate blend of operational expertise with data collection, analysis, critical interpretation, and sharing [27]. Involving a different range of actors such as policymakers, humanitarian workers, social scientists, communities, and local institutions was highlighted as essential [24,28,29]. The disaster-oriented literature considers collaboration between behavioral scientists, in particular psychologists, and communities as essential, especially regarding mental health interventions, showing the connection between social science and community engagement [28]. 

However, it was indicated that there might be issues with this kind of collaboration due to potential discrepancies in viewpoints, views, and expectations. Interagency competition can strain relationships and make them confrontational. Priorities also need to be established while taking into consideration power dynamics and inherent inequalities [11,15]. Regarding this, Lee (2008) argues that among the biggest challenges—besides the lack of resources, political will, or skilled staff—is the effective integration of established perspectives and methods for use on the ground, such as participatory methodologies or other examples detailed in the previous section [15]. In addition, it was highlighted that community priorities may be different from those of the experts, especially for professionals unfamiliar with local conditions. During conflict, it is also unlikely that communities and experts will have the same priorities [11]. Moreover, the analyzed literature did not provide any further information about the relevant contribution and engagement of the different social science approaches (e.g., anthropology versus sociology versus political science versus economics), even though social scientists from a wide range of disciplines are involved in emergencies. In addition, only one of the articles added a reflection on the possible diverging epistemological or ontological views across these scientific fields that may influence CE approaches. Panter-brick (2022) addressed the issue raised by research in the humanitarian sector, particularly about what constitutes “rigors evidence”, as the apparent hierarchy of evidence is consistently contested. Instead of creating evidence hierarchies, the author stressed the need to work with a network of evidence [22].

When it comes to coordination between all the humanitarian actors, including social scientists, a few articles emphasized the problem of dispersed efforts that result from the many different organizations and entities on the ground [21,25]. Fragmented information and limited engagement and coordination on resources and programs on disaster preparedness are major obstacles to effectively and efficiently planning for and responding to diverse population needs [30,31]. This also includes a lack of consistency in defining and naming community engagement. Within the humanitarian field, community engagement is associated with various names and expected functions, such as accountability to affected populations (AAP), communicating with communities (CwC), community engagement and accountability (CEA), and communication and community engagement (CCE) [31]. A possible solution presented to this observed lack of coordination is the creation of cluster approaches and network subgroups in the affected area [31].

### 3.3. Building Partnerships with Local Social Scientists, Networks, and Institutions

The reviewed literature emphasized the importance of ethical conduct when applying social-science-informed research methods among vulnerable, disaster-affected groups, including its dependence on understanding cultural and contextual elements, the timely dissemination of findings, and data sharing [32]. Karadag et al. (2021) notes that the engagement of local social science researchers and institutions and acknowledging local knowledge and experience is vital for better research and practice outcomes [16]. The advantages of building partnerships with local institutions includes ensuring issues are focused on recipients’ needs and addressing and understanding better multiple key sociocultural and contextual elements [33]. In addition, community engagement in some situations was emphasized as key to resolving ethical and logistical concerns that social scientists were not able to resolve [22]. The literature notes that attempts to access funding and define post-disaster strategies without considering the information provided by communities before and during a crisis can lead to a lack of collective accountability and poor coordination, leaving gaps and creating a duplication of efforts in operations [18]. In general, the absence of reliable funding for a mainstreaming approach often results in CE being included as separate activity instead of an integrated one. This approach leads to projects incorporating CE activities without adequate coordination, resulting in duplicated efforts, causing confusion among users, and limiting local participation [25].

Furthermore, the literature highlights pre-existing relationships for quicker implementation of programs [18]. The collaboration between national NGOs, as mediators of funding, with community-based organizations has been proven to be beneficial for CE work, as local organizations have access to existing communication pathways [25]. 

Yet, there is no mention of how social sciences can help to support engagement with these pre-existing relationships. A further standardization of the humanitarian response focused on the local priorities rather than each individual sector is one of the suggestions made. This is specifically relevant to the current discourse on the need to invest in preparedness and better mitigation of humanitarian crisis at the community level. According to Holloway and Fan (2018), this approach allows for a more effective collective advocacy in a response where the humanitarian actors are not entitled to make decisions themselves [19]. But again, the role of social sciences in this is also not made explicit here.

### 3.4. Localization of Decision-Making and Instrumentalism

There is a lot of information about power relationships, but social science approaches to address or understand these issues are not clearly noted. The literature emphasizes that programs that do not involve beneficiaries meaningfully may discourage active and sustainable participation later [14]. Humanitarian organizations are reported to lack a mechanism to “close the feedback loop”, meaning they lack communication back to the affected people after receiving community feedback [17]. Even if the community participates and is engaged in the humanitarian response, there is no curation of the data or analysis to improve the response [18]. Weine et al. (2021) highlighted that investing in building the local partners’ capacity is essential to improving social science research implementation and promoting scaling up and dissemination [34]. 

On the other hand, it is noted that building new partnerships may not be essential, depending on the community’s resources, as community engagement in disaster events can be integrated into existing social, economic, or health-related activities just as well or even more effectively [29]. For example, “post-disaster social entrepreneurship” (PSDE) or offering opportunities to locals to organize a sustainable post-disaster recovery, in the short and long term, has shown good results in engaging the community into its physical and economic infrastructure recovery where the local government and NGOs’ are weak, have low resources, or have a limited scope [35,36]. However, community engagement is argued to not be a one-time effort but a continuous, collective learning process, including iterative interactions that must be created far before an emergency event and endured in the aftermath. Consequently, institutional commitment, specific funding allocation, specialized staffing, and resources to enable participatory community planning are necessary for effective community participation [29]. Collective approaches for community engagement can improve the quality of the aid provided but also support a better acceptance of humanitarian actors by the community, hence enhancing humanitarian access [18]. This, presumably, also extends to social science actors, but this is not made explicit in the reviewed articles.

Connected with the issue of unequal power distribution, community engagement is often represented as an instrumental activity, and beneficiary involvement is typically restricted to needs-only data collection in emergency situations and only under conditions imposed by international partners [15]. For instance, the institutional cultures often see communities as implementing partners for international actors [21]. Therefore, greater support is needed to bridge local and national organizations’ capacities in a way that community organizations can be part of the national coordination framework [25]. Some studies envisioned community engagement to be more transformative with a call for better integration of communities, such as in the study of Panter-Brick et al. (2020), where local ownership was fostered through a discussion of the issues of research design and study implementation with research partners and communities [37]. In another study, it was remarked that local communities that welcome international organizations into their midst had the right to learn whether programs worked or did not, and they had a timely opportunity to contribute to their improvement [22].

## 4. Discussion

Community engagement plays a vital role in ensuring the effectiveness and sustainability of humanitarian interventions while fostering collaboration and participation. Social science approaches can help the integration of CE activities to improve the sustainability of interventions. The examples in Box 1 show how social science approaches can support CE, consider the cultural and needs assessments, stimulate community participation, and integrate community members into the delivery of an intervention. Studies suggest that tools such as cultural and needs assessments, rapid participatory approaches, and capabilities mapping are often used, and these are firmly based on social scientific insights and expertise. Techniques to involve community members in research studies, including focus groups and qualitative research techniques that effectively solicit community feedback, are essential to an effective response using a “collective approach” [18]. However, despite the humanitarian action’s commitment to implement locally relevant interventions, the relevance of social sciences in settings of conflict and natural disasters remains undiscussed compared with other fields, such as epidemic preparedness [4].

Coordination and collaboration between a different range of actors, including international, national, and local organizations, policymakers, humanitarian workers, social scientists, and the community, was emphasized as essential in various sources [24,25,26,27]. Coordination between all actors is essential to avoid a duplication of efforts. However, this collaboration comes with challenges such as discrepancies in viewpoints, approaches, priorities, and expectations, leading to a dispersion of efforts and inconsistent and ineffective outcomes [18]. In addition, the literature only marginally touches upon the possible diverging epistemological or ontological views that undergird the social science discipline used in emergency settings. 

Building partnerships and networks between social sciences and institutions was highlighted as beneficial in addressing the community need, understanding the context, and improving research and practice outcomes. However, the current practices of subcontracting agreements that see local actors as implementing partners and CE as an instrumental approach fail to give equal partnership to communities [21]. More robust support and better allocation of funds to CE activities can be transformative for integrating communities into the humanitarian response. Still, this can only be done if the community can contribute to the programming design and dissemination, and, as observed by Andrulis et al. (2011), effective community participation should be a continuous, collective learning process that includes iterative interactions that must be established long before a disaster and maintained after that [31]. Yet, the role of social sciences in facilitating and supporting the relationships between the different actors in the humanitarian action of conflict and disasters remains unexplored. 

These findings indicate that social science has not been professionalized as an explicit knowledge valuable base for and referenced by practitioners with its own support mechanisms. This is not to say that social science techniques are not used within these contexts. Instead, social science techniques are subsumed and implied, informally present but formally absent. While technically present, the merit of social sciences as a theory-informed, critical perspective that may support a more transformative shift in power balance needs to be present.

## 5. Conclusions

Humanitarian settings of disasters and conflicts often present unique scientific challenges and conditions that distinguish them from standard research settings. To our knowledge, this is the first scoping review focusing on the relevance and integration of the social sciences for community engagement in these humanitarian contexts. Other reviews have been published; however, they focused either on community engagement or social disciplines in the humanitarian context but not on both [32,38].

While we looked at nearly a thousand published articles of possible relevance to this topic, we saw only a few studies explicitly addressing social sciences as contributing to community engagement, all in passing and focusing on technical aspects. This lack of explicit attention to the merits of a social science approach or the type of social science is even more pronounced in the grey literature. These results attest to the wide gap which initiatives like UNICEF’s SS4CE project try to fill. Based on the results, we can propose five general actions regarding the relevance and organization of social science for community engagement. 

First, the examples show that high-quality applied social science research through flexible methodologies is possible for humanitarian programming, and moreover, such research can provide methodological innovations for improving community engagement techniques among practitioners. We found that interventions using social science methods are more culturally acceptable and more inclusive. Studies suggest that tools such as cultural and needs assessments, rapid participatory approaches, and capabilities mapping are, in fact, used often, and these are firmly based on social scientific insights and expertise. Techniques to involve community members in research studies, including focus groups and qualitative research techniques that effectively solicit community feedback, are essential to an effective response using a “collective approach”, and they are very useful as tools to understand patterns in quantitative findings. Therefore, ensuring that these social science methodologies are systematically and rigorously standardized and mandated in humanitarian programming processes will increase the effectiveness of people-centered outcomes of humanitarian and development programs.

Second, improved collaboration between individuals in the field, humanitarian partners, and social scientists in academic environments was noted to promote an important blend of operational expertise. Interdisciplinary institutes, such as the World Association for Disaster and Emergency Medicine, are essential to promoting such cross-sectoral and cross-disciplinary work. At the same time, an awareness of the critical and transformative contributions of transdisciplinary and/or multidisciplinary social sciences is missing. Given the involvement of social scientists from a wide range of disciplines, more attention needs to be given regarding the possible diverging epistemological or ontological views that may emerge.

Third, humanitarian programs are not implemented in isolation but in a social arena where context, policy, and actors are all intertwined; thus, these political dimensions should be considered. While social sciences directly contribute to analyzing these political governance contexts, they are also affected by them. In addition, social sciences can provide local understanding and insights into recipients’ needs, enhance coordination ensuring the lack of duplication and dispersion of efforts [18]. Therefore, to be effective, social scientist must also be advocate for and claim their seat at the table. 

Fourth, community-based disaster management can only be possible by allocating enough resources, building on existing capacities and pre-existing relationships of affected communities, including social entrepreneurship, and providing capacities when necessary [16,36]. Funders should recognize the need to include communities in the coordination framework, not merely as implementing partners, and they should acknowledge the unique challenges faced in conducting social science research during crises and allocate funding accordingly [16,21,25].

Finally, social scientists have a crucial role in facilitating the systematic and ethical accountability of research and programmatic data to local actors in order to accommodate the different priorities of the communities and humanitarian actors, given the potential discrepancies and inherent inequalities cause by power dynamics present in these situations [11,15]. At the same time, especially within the engagement process, research data should be manageable for the affected communities [39]. Given the need of a timely and manageable and sustainable response, the role of social science innovation and entrepreneurship during disasters and conflict is an important area for further study.

In conclusion, social-science-based CE could be employed to create trust, negotiate ethical difficulties, address power dynamics, collect and give feedback on appropriate data, and ensure that research findings will assist communities affected by the humanitarian crisis. However, while social science research practices and tools contributed significantly and provided significant opportunities, this scoping review identified a major awareness gap in both the peer-reviewed and published literature regarding social science contributions to community engagement in the humanitarian contexts of natural disasters and conflicts. This gap is a need for a more explicit understanding of its contribution, particularly regarding the transformative power of more critical perspectives and challenges regarding integrating its contributions in the humanitarian field. In contrast to disease outbreaks, in disasters and conflicts, the role of social sciences in the literature remains implicit and presumed and is rarely made explicit. Clarifying the added value of social sciences is urgently needed to bring out its impact and professional identity, which is now subsumed in undefined practices. To achieve this, continued resources and investments are needed to promote multi- and transdisciplinary social science methodologies, improve the collaboration between social scientists and programmatic stakeholders, systemically address the role of social sciences in decision-making, and support accountability to communities involved.

## Figures and Tables

**Table 1:** Population, concept, and context (PCC) framework used in this scoping review.

PCC	Explanation
Population	Social sciences/social scientists working in the field of community engagement
Concept	Lessons on integration, expansion, or adaptation
Context	In humanitarian preparedness, response, and recovery from conflicts and disasters

## Data Availability

A summary of the data (selected articles for review) presented in this study is available in Table S1: Overview of selected studies, peer-reviewed, and grey literature.

## Supplementary Materials

The following supporting information can be downloaded at: https://www.mdpi.com/article/10.3390/ijerph20196856/s1: Table S1: Overview of selected studies, peer-reviewed, and grey literature [11,12,13,14,15,16,17,18,19,20,21,22,23,24,26,27,28,29,30,31,32,33,34,37,38].
