# Supplementary material for: The Integration of Social Science for Community Engagement in the Humanitarian Fields of Conflicts and Disasters: A Scoping Review
Source: Int J Environ Res Public Health. 2023 Sep 28;20(19):6856. doi: 10.3390/ijerph20196856 (PMC10573063; doi:10.3390/ijerph20196856)
Supplement: Supplementary file 1 [file ijerph-20-06856-s001.zip › ijerph-2499921-supplementary.pdf]

Table S1: Overview of selected studies, peer-reviewed, and grey literature.

#		Year	Title	Country	Study Design	Humanitarian Context	Summary of Findings
1	Bava et al. [27]	2010	Lessons in Collaboration, Four Years Post-Katrina	United States of America	Qualitative (secondary research: case study)	Disaster	Lesson learned include: the divergent perspectives in disaster mental health field; that more face-to-face introductions would have encouraged a wider community invitation to develop a collaboration. Outsiders would have had more opportunities to develop trust with local mental health and community-service workers and to assess how local needs might match outsider resources; The fundamental quality shared by all successful collaborations is that the participants listen to each other and that those people (often professionals) initiating collaborative conversations allow other participants (clients and/or community members) to change the course of a conversation as it develops; As in all human endeavors, timing was critical. It is possible that 2 years post-disaster was an especially difficult time for outsiders to attempt to partner with community groups.

2	Tuerk et al. [24]	2013	Forty days after the great East Japan earthquake: Field research investigating community engagement and traumatic stress screening in a post-disaster community mental health training	Japan	Mixed-method	Disaster	Participant engagement in the training forum was robust. Exposure to potentially traumatic events was high in this sample. Most people reported having experienced one potentially traumatic event prior to the most recent earthquake (52%), 24%reported two exposures, and 24% reported three or more exposures.
3	Margolin et al. [29]	2010	Earthquakes and Children: The Role of Psychologists With Families and Communities	China	Qualitative (Secondary Research)	Disaster	Sustaining and empowering natural care-giving systems in the family, school, and community are essential for earthquake preparedness and restoring these systems is indispensable to reducing post-earthquake effects in children. Psychologists can play active and important roles, as part of interdisciplinary teams, to build self-reliant and self-sustaining systems of care capacities within local communities, and to coordinate these local integrated systems with external humanitarian relief efforts when needed.
4	Weine et al. [34]	2021	Conducting research on building psychosocial support for Syrian refugee families in a humanitarian emergency	Istanbul	Qualitative	Conflict	They identified multiple challenges: Non-existent or weak partnerships geared towards mental, Health Research in a humanitarian emergency, Lack of familiarity with task-sharing, Insufficient language and cultural competency. Fit with families' values and demands, Hardships of urban refugees. In order to address these challenges they identified different research strategies. The strategies included: coalition building,<br>Building research capacity of local partners, Engaging refugee family experiences and perspectives, Flexible

							intervention design, Fostering evidence-based policy and program development for refugees.
5	Rivera-Holguìn et al. [20]	2016	A post disaster capacity building model in Peru	Peru	Qualitative (Secondary Research)	Disaster	The participatory workshops promoted community mobilisation and joint action in order to improve the living conditions of the population. The impact of the capacity building approach occurred not only on a personal level, but also at community level. This change translates into concrete actions, such as the improvement of public space. Thanks to community mobilisation and action, it is now possible to improve public spaces post-earthquake with collective initiatives from the community
6	Song et al. [14]	2013	Assessing the Impact of Violence and War on Youth in Low- and Middle-Income Countries	LMICs	Qualitative (Secondary research: commentary)	Conflict	Understanding the practical and ethical concerns of conducting research for youth exposed to violence and armed conflict can assist in producing studies that are powerful tools to advocate for those affected by trauma in low- and middle-income countries.
7	Karadag et al. [16]	2021	Challenges and lessons learned in mental health research among refugees: a community-based study in Turkey	Turkey	Qualitative (secondary research: 'lesson learned')	Conflict	The challenges identified by the researchers included: working with a highly traumatized population, legal status, lack of data, and high mobility of refugees, Safety issues and willingness to participate, Referral for services, cultural barriers, resentment from host communities, language. The strategies used to adress these challenges included: training of data collectors, cultural

							considerations and gender-sensitive approach, sampling methodologies, community participation and pilot testing, and safety considerations.
8	Mistry et al. [30]	2021	Introduction to collection: confronting the challenges of health research in humanitarian crises	Collection of articles spanning 27 countries, and covering a broad range of humanitarian crises	Qualitative (secondary research: articles collection)	Both (disasters and conflicts)	-need of research: First and foremost, there is a clear need for humanitarian health research. Several authors noted the limited evidence base in their area of study and all the studies in tris collection aimed to address an evidence gap or need. Partnership with humanitarian actors, including local governments, local and international NGOs, and UN agencies, was found to be a critical strategy in the collection.
9	Vega Ocasio et al. [26]	2020	Conducting an immersive community based assessment of post-hurricane experience among Puerto Ricans: lived experience of medical ecology in an environmental	Puerto Rico	Qualitative (Secondary research)	Disaster	Lesson 1: using the critical medical ecological model helped organize our work and response. Lesson 2: incorporation of participation and methods that prioritize authenticity. Investigators recognized the need to approach our research participants as collaborators and to strive toward building and relying upon authentic partnerships to collect and analyze data. Lesson 3: understand the trauma experience and incorporate study methods sensitive to it. Given the massive trauma experienced by Puerto Ricans in this period, and their witness to that suffering, they needed to prioritize

			disaster and migration				understanding such experiences and incorporate it into our approach
10	Safarpour et al. [32]	2020	Challenges and barriers of humanitarian aid management in 2017 Kermanshah earthquake: a qualitative study	Kermanshah province in west of Iran	Qualitative	Disaster	Managerial barriers:<br>-lack of public and organizational education -Command and coordination: the multiplicity of the organizations responsible for the disasters is considered as a serious challenge for managing humanitarian aids and donations.<br>-Communications and information: They were dissatisfied with the weak performance of the media and lack of control on several social networks.<br>- Lack of rules and supervising system. Structural barriers<br>- Security - Assessment: Lack of assessing the needs and capacity of the area is influenced by two main challenges reported by the participants.<br>-Providing system: reserving and distributing the humanitarian aids<br>-Cultural setting: They emphasized that lack of community trust in GOs versus the excessive trust they had in celebrities and renowned persons as one of the main challenges. The responsible GOs and the NGOs were inattentive to people's culture and customs, leading to some problems in managing humanitarian aids.

11	Jahangiri et al. [38]	2021	A comparative study on community-based disaster management in selected countries and designing a model for Iran	Iran	Qualitative (secondary research: systematic literature review)	Disaster	Various participatory actions in mentioned seven selected countries were illustrated to show the different level of participation (in the process of planning, policy making, organizing, coordination and control). Based on the results, a model has been designed and proposed in order to make the community based disaster management more feasible in Iran. One of the characteristics of this model is the involvement and participation of the community through designing "Strategic Planning Councils" for guiding the stakeholders' participation. The role which strategic councils play in this level is more of consultation and the emphasis of this model is on people participation on the local level in villages and neighborhood participation in the cities.
12	Cueto et al. [21]	2015	Community Participation and Strengthening in a Reconstruction Context After a Natural Disaster	Peru	Qualitative	Disaster	The analyzed results herein revolve around three axes that emerged from the work sessions and that are linked with the concepts of enhancement and community participation in the framework of a process of reconstruction post-disaster: (1) the felt and prioritize needs following a disaster, (2) the relationships with power and leadership within the communities, and (3) the characteristics and reaches of community participation in the areas of study.
13	Lee [15]	2008	Local perspectives on humanitarian aid in Sri Lanka after the tsunami	Sri Lanka	Qualitative	Disaster	• This study found that rapid participatory approaches can be used to obtain beneficiary feedback in post-disaster settings to guide programme planning<br>• Satisfaction of beneficiary needs is essential<br>• An understanding of the context in which aid is delivered is essential. Failure to do so risks exacerbating

							pre-existing tensions or creating new ones.<br>• Beneficiary accountability requires meaningful community engagement, good handling of community relations, and sensitive matching of aid to perceived needs.
14	Padmavati et al [23]	2020	Learnings from conducting mental health research during 2004 tsunami in Tamil Nadu, India	India	Qualitative	Disaster	Despite limited data, conclusions were made regarding how to improve psychosocial aid in the future, including educational materials for aid workers and the public, reaching out<br>to communities with higher rates of mental illness, preparation for region specific cultural consideration, as well as dissemination of information for staff on how to deal with<br>vicarious trauma.
15	Andrulis et al. [31]	2011	Integrating Racially and Ethnically Diverse Communities Into<br>Planning for Disasters: The California Experience	United States of America	Qualitative (secondary research: literature review)	Disaster	Individual-level barriers:<br>• Socioeconomic factors.<br>• Trust; perceived fairness of government.<br>• Culture and language. Institutional-level barriers:<br>• Lack of funding for diversity initiatives.<br>• Limited knowledge about diverse communities.<br>• Limited collaboration with communities.<br>• Integrating social and economic circumstances of communities in emergency planning and response.

16	O'Sullivan et al. [12]	2014	Use of the Structured Interview Matrix to Enhance Community Resilience Through Collaboration and Inclusive Engagement	Canada	Qualitative	Disaster	The SIM is a useful tool to implement these strategies as it facilitates widespread interaction amongst the group, and empowerment and recognition of each participant's voice, while exchanging information and developing plans for collaborative action through the three steps. Awareness and common ground are important capacities for community resilience and promoting adaptive response to disasters. They are also key mechanisms for fostering action during community development initiatives. Common ground does not develop instantaneously, but rather it is the product of an iterative cycle of engagement, information exchange, networking, an empowering climate and inclusion in collaborative activities.
17	Panter-Brick et al. [37]	2020	Measuring the psychosocial, biological, and cognitive signatures of profound stress in humanitarian settings: impacts, challenges, and strategies in the field	Jordan	Qualitative (primary research)	Conflict	• They learnt that high-quality scientific research is feasible, and can be useful and ethical, in humanitarian settings: with the support of a local research team and active engagement with their community<br>• Second, we found young people in the context of a humanitarian crisis to be engaged, motivated, and informed regarding the value of scientific research seeking to determine effective means of supporting them in dealing with the profound stress of conflict and displacement.<br>• Third, we learnt that sustained partnerships between scholars, humanitarians, funders, and beneficiaries across multiple locales produces a strong sense of research ownership – back-and-forth discussions of complex challenges and heterogeneous findings help grow the research agenda.<br>• Finally, we learnt that opportunities for frequent, meaningful conversations are strongly needed– and that

							serious dedication to one's work does not preclude dialogue and humour.
18	Banatvala [28]	2000	Public health and humanitarian interventions: developing the evidence base	Not specific	Qualitative (secondary research: case analysis)	Both (disaster and conflict)	People caught up in complex emergencies are often highly vulnerable and may have been severely abused. The key elements of an ethical approach are maximising benefit and<br>minimising harm, obtaining informed con- sent, ensuring confidentiality, and treating individuals with appropriate clinical care and dignity. Improved collaboration between individuals in the field and those in the academic environment can help promote an appropriate blend of operational expertise with the collection, analysis, critical interpretation, and dissemination of data. Traditional methods of continuing<br>professional development through printed media, conferences and workshops, and training courses are of value but have inherent limitations. Improving opportunities and funding to facilitate linkages of academic institutions and non-governmental

							organisations and to establish mechanisms for disseminating and debating key findings with relevant stakeholders— donors, host governments, service providers, and, wherever possible, representatives of affected communities—will increase the likelihood of benefits being derived from earlier investments in research and evaluation
19	Guha-Sapir [33]	2020	Challenges in public health and epidemiology research in humanitarian settings: experiences from the field.	India, Phillipines, Nepal	Qualitative (secondary research: commentary)	Disaster	• Contingency plans for dealing with logistical and administrative challenges help to anticipate potential barriers and foster timely problem solving for those that are unforeseen.<br>• Choosing data collection methods and sources to sidestep concerns associated with displacement and destruction ensures access to study subjects. It also mitigates concerns<br>of bias introduced into standard sampling frames used in population surveys.<br>• Research teams must work closely with local teams, consult with them from the start, and share research benefits fairly. This is not only in the interests of equity, but it protects the research outputs from embarrassing oversights and enriches the results.<br>• Further, researchers in humanitarian settings must weave through complex political and cultural labyrinths, requiring a clear idea of the agendas being promoted by different stakeholders and the potential impact these narratives have on the dissemination of research.<br>• Understanding cultural and circumstantial factors surrounding research,<br>• Disseminating results in a timely manner, and sharing

							data are critical for the ethical conduct of research amongst vulnerable, disaster-affected populations.
20	Durrance-Bagale et al. [11]		Community engagement in health systems interventions and research in conflict-affected countries: a scoping review of approaches	World	Qualitative (secondary research: scoping review)	Conflict	Community engagement in identifying and setting priorities, decision-making, implementing, and evaluating potential solutions helps people share their views and encourages a sense of ownership and increases the likely success of healthcare interventions. However, engaging communities can be particularly difficult in conflict-affected settings, where priorities may not be easy to identify, and many other factors, such as safety, power relations, and entrenched inequalities, must be considered. Involvement of community leaders (trust and accessibility), contextualisation (e.g. ensuring that conflict, displacement, and other community context is considered), equity (ensuring everyone has opportunities to share their views), transparency (providing a forum for feedback) and autonomy (community bottom-up leadership rather than top-down whenever possible). One issue is that community engagement is often talked about but is framed as a consultative process rather than an attempt to empower communities, with the aim of putting final decision-making in their hands

21	Panter-Brick [22]	2022	Energizing partnerships in research-to-policy projects	World	Qualitative (secondary research:	Conflict	Approaches that foster synthetic knowledge and creative partnerships through biocultural and people-centered research are not easy to implement and certainly unusual in conflict zones. Two benefits of biocultural research are to create room for synthetic conversation with diverse audiences and creative ways of working with communities. Biocultural research on refugee well-being helps to reach a diverse range of interlocutors: scholars, practitioners, policymakers, funders, and civil society actors who work with crisis-affected people. Importantly, biocultural work can be rooted, creatively, in practices that respect and value our shared humanity. Community-empowerment tool kits proved insufficient to tackle entrenched power hierarchies, lack of trust, and conflict over material resources; these effectively undermined a project to address health inequities and build social cohesion among refugees and host populations. Embedding research within people-centered partnerships, equitably, is an iterative process that traces an arc from project design to dissemination.
22	CDAC Network [18]	2017	The role of collective platforms, services and tools to support communication and community engagement in humanitarian action	World	Policy paper	Both (disaster and conflict)	Integration of communication and community engagement with feedback from population is key to the response mechanisms. Dedicated resources (either financial and human) to communication and community engagement is more effective where added onto existing staff responsibilities. International organization should recognize and use local existing communication and community engagement mechanisms. Capacity building in communication and community engagement strategies as an approach in preparedness planning

23	Barbelet [17]	2020	Collective approaches to communication and community engagement in the Central African Republic	Central African Republic	Report	Conflict	There needs to be more dissemination of information to country-level actors involved in collective approaches, including Humanitarian Coordinators, cluster leads, NGO coordinators, and coordinators of the collective approach. Guidance is needed for the agency that takes a lead in driving the collective approach, for example UNICEF. This should include information on how best to manage hosting the collective approach in terms of reporting lines, budget responsibility, standard operation procedures for decision-making and fundraising guidelines.
24	Holloway and Fan [19]	2020	Collective approaches to communication and community engagement in the Central Sulawesi response	Indonesia	Report	Disaster	The response to the Central Sulawesi earthquake, tsunami (...) demonstrates that a collective approach is possible, but it does not guarantee good CCE. Likewise, CCE can happen without a collective approach, but it did benefit from it. In this response, the communication of important information to affected communities did not really improve, nor was the response modified significantly based on their feedback, though likely more than if there had not been a collective approach.
25	Yonally et al. [13]	2021	Review of the Evidence Landscape on the Risk Communication and Community Engagement Interventions Among the Rohingya Refugees to Enhance Healthcare Seeking Behaviours in Cox's Bazar	Bangladesh	Report	Conflict	The risk communication aspect of RCCE, and content of messaging relating to risk, was generally considered to be appropriate in Cox's Bazar. However, what was still lacking is two-way engagement that provides the refugee population with the opportunity to engage with the material provided, ask questions, and contribute their own experiences, concerns or ideas.
